# Communication in Oncology Outpatient Clinic Settings: Congruence of Quality of Life Assessment between Patient-Physician and Patient-Caregiver Dyads

**DOI:** 10.1155/2022/5463896

**Published:** 2022-08-25

**Authors:** Chia-Chun Tang, Chen Hsi, Wu Wei-Wen, I-Ni Tsai, Tsai Jaw-Shiun

**Affiliations:** ^1^School of Nursing, College of Medicine, National Taiwan University, Taipei, Taiwan; ^2^Department of Nursing, National Taiwan University Hospital, Taipei, Taiwan; ^3^Teaching Chinese As A Second Language, National Taiwan University, Taipei, Taiwan; ^4^Department of Family Medicine, College of Medicine, National Taiwan University, Taipei, Taiwan; ^5^Department of Family Medicine, National Taiwan University Hospital, Taipei, Taiwan

## Abstract

**Objectives:**

The aims of this study were to investigate the congruence of HRQOL reports between patient-physician and patient-caregiver dyads and to determine the association of variables, if any, with the congruence between dyads.

**Methods:**

This correlational study with a cross-sectional design first approached physicians who provided care for patients with advanced cancer at the participating institution. Then, participating physicians' patients and their caregivers were recruited. All participants were required to independently fill out an HRQOL questionnaire during their outpatient visits. Descriptive statistics, weighted kappa, Wilcoxon signed-rank test, and linear regression were employed for data analysis.

**Results:**

A total of 52 patient-physician and 27 patient-caregiver dyads were examined. Patients suffered from considerable problems in all three HRQOL domains: symptom, functioning, and overall HRQOL. The patients' level of agreement was moderate with the caregivers and fair with the physicians. A significant relationship was found between several patient-related variables and disagreement.

**Conclusion:**

These patients with advanced cancer experienced a compromised HRQOL, warranting immediate attention. When there are barriers to obtaining a patient's self-report, clinicians may consider caregivers as a reasonable source. Patients with special characteristics need additional attention because their problems may be at a greater risk of being overlooked.

## 1. Background

Health-related quality of life (HRQOL) has been an important concept in the medical field since the 1970s [1]. Although the definitions of HRQOL may vary slightly across studies, most experts agree that HRQOL contains several dimensions, including symptoms, functional status, and general health perceptions that describe a full range of variables related to patient outcomes [2]. Mounting evidence has shown that HRQOL is directly associated with mortality and cancer-related outcomes, such as the rate of cancer recurrence [3, 4]. In terminally ill population, HRQOL has been measured to reflect quality of palliative care and the impacts of symptoms and interventions [5–7]. While individuals are the most ideal and reliable source of their health-related quality of life (HRQOL), it is common to determine the HRQOL of terminally ill patients based only on the evaluation of their proxies owing to the patients' deteriorating physical and psychological conditions. Examining the extent to which these proxy raters can be relied on and the factors that may affect their evaluation is imperative. In addition, exploring the degree of congruence between patient's and healthcare providers' evaluation provides clues to the communication quality.

Studies focusing on patient-caregiver agreement of the evaluation regarding various domains of HRQOL have increased significantly over the past two decades. The results have been mixed—several studies have suggested that caregivers' report of cancer patients' HRQOL is close to patients' self-report; [8–12] however, a couple studies have reported different evaluations between the caregivers and the patients [13, 14]. Although the agreement levels have been varied, many studies agree that caregivers generally overestimate the patients' problems [8, 11, 13, 15]. Researchers have found that healthcare providers have different tendencies as compared to caregivers; healthcare providers' perspectives deviated more from the patients', [16–18] and often underestimated their problems [19–23]. Patient factors such as age and severity of symptoms are all possible influences that affect the agreement between proxies [15, 17, 18, 20, 23, 24]. As the evidence regarding the level of congruence between patients and proxies has been developing, more questions have emerged. For instance, although symptoms are “perceived indicators of change in normal functioning as experiencing by patients” [25], symptom report and evaluation is essentially a communication process shared among patients, caregivers, and healthcare providers. Thus, the discrepancy between evaluations is not only associated with how well proxies can capture patients' feelings but also indicates the quality of their communication. However, most relevant studies have not addressed any issues regarding this communication. Study designs that specify the timings between symptom evaluation and the related communication can provide hints regarding the quality of communication based on symptom agreement.

Accordingly, the current study was constructed in outpatient department (OPD) settings in Taiwan to (1) examine advanced cancer patients' various domains of HRQOL as reported by patients, caregivers, and physicians, (2) to investigate the congruence level of HRQOL reports between patient-physician dyads and patient-caregiver dyads, and (3) to determine if any patient variables are associated with the congruence level between dyads. Collecting data in OPD settings allowed us to emphasize the timings between evaluation and communication which was described in the following method section.

## 2. Methods

### 2.1. Study Design

This was a correlational study with a cross-sectional design. Physicians taking care of patients with solid tumor at a medical center in Northern Taiwan were first recruited through personal contacts. A research assistant then consulted a participating physician to identify potentially eligible patients. The inclusion criteria for the patients included consulting a participating physician, being diagnosed with advanced solid cancer (TNM stage III or IV), aged 20 years or older, and being able to communicate in Chinese or Taiwanese. However, patients who did not experience any symptoms or were hospitalized at recruitment were excluded. Because the maturity may affect one's ability to communicate and self-evaluate, we set the age limit to focus on adult patients only. According to the local law, twenty years old is the lower legal age of adulthood.

Simultaneously, adult main caregivers who accompanied the participating patients to their OPD visits were also invited to participate in the study. Participating patients and caregivers were required to independently fill in questionnaires shortly before or after the OPD visits. Physicians were required to complete the same questionnaire for each participating patient immediately after their OPD discussion. The data collection period was from December 2018 to December 2019. This study was approved by the National Taiwan University Hospital Human Subjects Office Institutional Review Board (201807052RINC, Sep, 2018). All participants provided written consent.

### 2.2. Instrument

The European Organization for Research and Treatment of Cancer quality of life questionnaire (EORCT-QLQ C30, Taiwan Chinese version) is a 30-item questionnaire evaluating cancer patients' HRQOL according to three domains: symptom, functional impairment, and the overall HRQOL. While items in the overall HRQOL domain are rated on a 7-point Likert scale, other items are rated on a 4-point Likert scale. The final scores for each domain are standardized and transformed to a range of 0–100. Higher scores indicate better conditions in HRQOL and functioning domain but worse problems in the symptom domains. The EORTC QLQ-C30 Taiwan Chinese version has been tested and shows good reliability and validity [26, 27]. In this study, the Cronbach's alpha for the responses of patients, caregivers, and physicians was 0.84, 0.92, and 0.93, respectively.

### 2.3. Statistical Methods

Descriptive statistics were used to analyze the demographic data and the score for each questionnaire item. Then, the score of each item was further compared to a clinically significant value to calculate the level of symptom burden [28]. The patient's level of symptom burden was the number of items that they rated as equal to or greater than the clinically significant value.

The congruence between dyads were analyzed from individual and group aspects. Weighted kappa was employed to assess the data from the individual aspect as to what categorized congruence into six levels: [29] no agreement (*k* = 0), none to slight (*k* = 0.01–0.20), fair (*k* = 0.21–0.40), moderate (*k* = 0.41–0.60), substantial (*k* = 0.61–0.80), and almost perfect (*k* = 0.81–1.00). A sample size of 32 and 20 for patient-physician and patient-caregiver dyads, respectively, were determined based on Bujang et al. 's guideline of the kappa agreement test [30]. We further calculate sum score differences of each dyad (physician or caregiver score minus patient score) to reflect the proportion of complete agreement (score = 0), overestimation (score > 0), or underestimation (score < 0). With regard to the group aspect, the Wilcoxon signed-rank test was applied [23].

To explore whether there was any variable associated with incongruence in HRQOL evaluation, linear regression was performed. The dependent variables, incongruence in each domain, were the total sum score difference of each domain. The independent variables that could potentially affect congruence were identified through a literature review. Software such as IBM SPSS 21, RStudio, and Octave were used for statistical analysis and to generate figures.

## 3. Results

A total of 52 patient-physician and 27 patient-caregiver dyads were included in the analysis. Among the eight physicians approached, six agreed to participate, including five males and one female (mean age = 42.67; SD = 5.5). Their average years of practice was 13.5 (SD = 3.8), either as medical oncologists (*n* = 3) or hospice specialists (*n* = 3). One hundred and twenty-four patients consulting the six participating physicians were approached, and 66 patients (53%) agreed to participate. Finally, 52 patients (78.8%) with 10 different types of cancer diagnoses completed the study (mean age = 61.6, SD = 12.1). The majority were male (80.8%), married (73.1%), and unemployed (55.7%). Thirty-five (67%) were recruited from the oncology OPD and 17 (32.6%) from the hospice OPD. Twenty-seven caregivers of the participating patients, including 24 females and 3 males, agreed to participate in the study. Their relationships with the patients were of partners (*n* = 16, 59.3%), children (*n* = 8, 29.6%), parents (*n* = 2, 7.4%), and siblings (*n* = 1, 3.7%). Their mean age was 55.2 years (SD = 9.3).

Based on the patients' perspectives, the top four symptoms that received the highest median scores were fatigue, appetite loss, insomnia, and financial difficulties with medians of 44.4, 33.3, 33.3, and 33.3, respectively (Table 1). The median burden level was five, indicating that the majority of patients had at least five symptoms that reached clinical importance. The median scores for the social, physical, role, cognitive, and emotional functions were 66.7, 73.3, 83.3, 83.3, and 83.3, respectively. The patients' median score for the overall HRQOL was 50. Both physician and caregiver ratings for HRQOL (median = 41.7 for both groups) were lower than the patients' ratings. Physicians' and caregivers' evaluation of functions were similar to the patients' self-ratings, except for role function and emotional function that had medians of 66.7 and 75, respectively, for both physician and caregiver groups. While physicians believed that pain, insomnia, fatigue, appetite loss, and constipation were all considerable problems for patients (median = 33.3 for all five symptoms), caregivers' evaluations of symptoms showed that insomnia (median = 66.7) and fatigue (median = 44.4) were the two most severe symptoms. Similar to the patients, the caregivers also highlighted financial difficulties (median = 33.3); however, the physicians considered it to be a minor problem (median = 0).

### 3.1. Congruence of the Assessment of HRQOL between Dyads

The following paragraphs present the congruence of evaluation according to different dyads and aspects (i.e., individual and group).

#### 3.1.1. Patient-Physician Dyads

From the individual aspect, patients and physicians had a fair level of agreement on majority of the items. However, their HRQOL ratings for problems in physical functioning, fatigue, pain, and dyspnea reached moderate agreement. Patient and physician dyads had none to slight agreement on financial difficulties. Physicians underestimated the patients' HRQOL, problems in social functioning, fatigue, insomnia, and pain. However, they overestimated the problems in role, physical, and emotional functioning (Table 2). When assessing the congruence of symptom evaluation from the group aspect, patients and physicians had significantly different ratings for diarrhea and financial difficulties.

#### 3.1.2. Patient-Caregiver Dyads

From the individual aspect, patients and caregivers had a moderate level of agreement on the majority of items. Moreover, their ratings of HRQOL, physical functioning, fatigue, and constipation reached a substantial level of concordance. On the other hand, they had a fair level of agreement on problems in emotional, cognitive, social functioning, and financial difficulties. On exploring how the caregivers rated differently than the patients, it was found that the former tended to overestimate almost all items, except for problems in social functioning (Table 2). From the group aspect, patients and caregivers had significantly different ratings for physical functioning, role functioning, emotional functioning, and pain.

### 3.2. Factors Associating with Congruence Level

**Figure 1:**
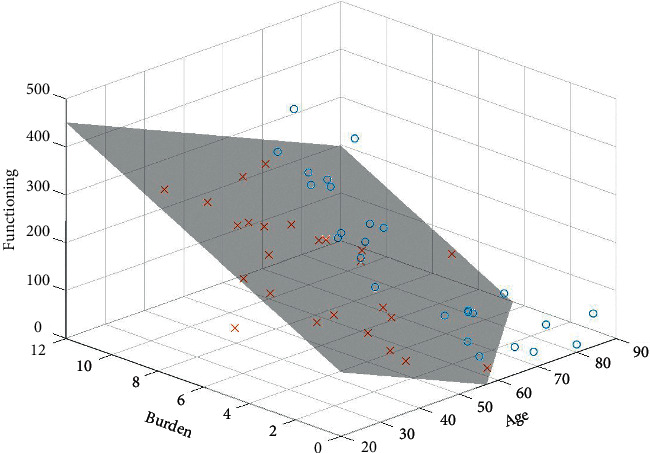
Relationship among functioning, burden level, age, and congruence of patient-physician symptom evaluation: the grey surface in the 3D regression plot depicts perfect congruence in symptom evaluation (patient-physician difference = 0); the circles and crosses demonstrate real data situated above and under the surface, respectively. The space above the surface means overestimation and the space under the surface represents underestimation. Category boundary for functioning impairment is 0–500 and 0–14 for burden.

**Figure 2:**
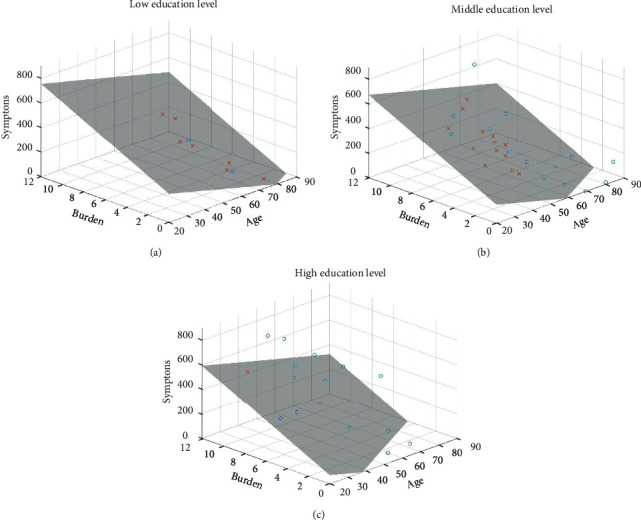
Relationship among symptom, burden level, age, and congruence of patient-physician functioning evaluation according to three education levels: the surfaces in the 3D regression plots depict perfect congruence in functioning evaluation (patient-physician difference = 0) according to three education levels: (a) low, (b) middle, and (c) high; the circles and crosses demonstrate real data situated above and under the surface, respectively. The space above the surface means overestimation and the space under the surface represents underestimation. Category boundary for symptoms is 0–900 and 0–14 for burden.

For patient-physician dyads, we found statistically significant relationships between patient-related variables and congruence in evaluating all three domains. Age, functioning status, and burden level together explained 20.3% of the variance in the congruence level of the patient-physician symptom evaluation (*P*=0.005). Patients with better function, higher burden, and younger age were more likely to be underestimated by the physicians in terms of their symptoms (Figure 1). Age, education, symptom severity, and burden level together explained 32.4% of the variance in the congruence level of patient-physician functioning evaluation (*P*=0.001). Patients with more severe symptoms, heavier burdens, and younger age were more likely to be underestimated by the physicians in terms of their functioning impairment. Furthermore, physicians were more likely to underestimate functioning status in patients with low educational levels, compared to those with higher educational levels (Figure 2). Functioning status and HRQOL explained 20.1% of the variance in the congruence level of patient-physician HRQOL evaluation (*P*=0.002). Patients' HRQOL were more likely to be underestimated by the physicians if they had poorer HRQOL and more severe functional impairment. For patient-caregiver dyads, caregivers were likely to underestimate the HRQOL in patients with poorer HRQOL and burden level. The HRQOL and burden level explained 23.1% of the variance in the congruence level of patient-caregiver HRQOL evaluation.

## 4. Discussion

This study examined the congruence level of symptom evaluation between patient-physician and patient-caregiver dyads during their OPD encounters. With respect to this study's first aim, we found that the participating patients experienced considerable symptoms and a compromised HRQOL. Compared to similar groups of patients in other reports, [31] the patients in this study experienced poorer conditions in the HRQOL, physical functioning, social functioning, fatigue, nausea and vomiting, pain, appetite loss, and financial difficulties. In a similar vein, the majority of these patients' ratings of physical functioning, fatigue, nausea and vomiting, and financial difficulties have been considered clinically meaningful [32]. Symptoms of clinical importance are changes or difficulties that cause worries, limit daily life functioning, or need help, and thus merit attention in the clinical discussions. This group of patients will benefit greatly from an in-depth symptom discussion and thorough management, including options of palliative care.

Although the physicians were inclined to give indiscriminate attention to all symptoms, a few that caused more distress than the others did stand out in patients' and caregivers' minds. In addition, physicians did not recognize the clinically significant financial problem identified by both patients and caregivers. It may be associated with the general impression of Taiwan's healthcare system (National Health Insurance) that promises equal access to healthcare for all the citizens and reaches 99.6% of the population [33]. Clinicians should be more sensitive to the scope of the problems faced by the patients and prioritize the management plan. For example, in Taiwan, where each OPD discussion is usually completed within 5 to 20 minutes, using symptom or HRQOL questionnaires may help pinpoint the most distressing problems quickly [34].

With regard to the second aim, individual aspect was inspected to reflect patient-centered situation and group aspect was examined for systematic bias [35]. From the individual aspect, while results from some studies showed moderate agreement [18, 21, 23, 36], our results indicated slight to fair agreement between patient-physician dyads which was lower than patient-caregiver dyads [16, 22]. The discrepancies in agreement levels across studies may be affected by patient conditions or demographic factors. For example, most of our subjects experienced heavy symptom burden and were predominantly male. It is necessary for the future studies to explore the effects of symptom severity or gender, if any, on symptom report or communication. The physicians' evaluations were more accurate in concrete and obvious items and the overall HRQOL. However, their evaluations were suboptimal in areas that were more concealed, even when the evaluation was done immediately after the relevant discussion. These results demonstrate outcomes that are in line with the previous studies' findings [15, 18, 21, 37]. There is an evident communication gap, and clinicians need to pay special attention to these hidden problems. Our study also confirmed the established evidence: physicians tend to underestimate the severity of most symptoms, but at the same time, overestimate patients' functional impairment [19, 38]. The percentage of completed agreement was lower in our study (∼17%–46%) than the others (∼78%–93%) [20, 23]. This may be due to the difference in the definitions of “completed agreement.” While most studies have used a 4-point Likert scale to measure symptom severity, studies with a higher percentage of “complete agreement” defined it as a score difference smaller or equal to one. This definition can generate approximately 62% of “complete agreement” if raters (e.g., physician) assigned random scores. This fact calls for more discussion and consideration to determine the definition and clinical meaning of “completed agreement.”

The results revealed that the caregivers could be a reliable source of patient information as they showed moderate to substantial level of agreement with patients on most items; however, they tended to systematically overestimate the patients' problems. Similar trends have been observed in previous reports [8, 9, 12]. Although the rationale for this phenomenon is unclear, caregivers' own concerns and experience of the disease (e.g., pre-loss grief symptoms, caregiver burden) may be associated with the overestimation. Moreover, the perspectives that proxies take may significantly affect their ratings. Pickard and Knight (2005) proposed that proxy raters may rate based on patient's view or their self-imaging view. A previous study has pointed out that caregivers tend to overestimate when they presume a self-imaging view [39]. There is little research exploring healthcare providers' perspective-taking when evaluating patient status. Whether perspective-taking affects the healthcare providers' evaluations and if they are aware of it are important issues to be explored. From the group aspect, while previous studies have observed statistically significant differences between patient and proxies in all HRQOL domains, [19] we found several differences reaching statistical significance. This may be caused due to the study's small sample size making it difficult to detect systematic differences.

Finally, for the third aim, our results corroborate previous findings that age [24, 40], symptom severity [15, 20, 24], and functioning status [15, 18, 23] are significant predictors of disagreement. In addition, we found that education may play a role in disagreements. While studies have pointed out that it is harder for healthcare providers to accurately reflect patient-perceived situations when the patients' symptoms are between moderate to severe [18, 23], our analysis further specifies its direction: when patients' problems are more severe, the likelihood of the healthcare providers to underestimate their problems increases.

### 4.1. Study Limitations

Some limitations warrant consideration. The sample size was small which prohibited us from using more advanced statistical methods, such as further grouping patients based on special characteristics. We also conducted a small poll for physicians and caregivers; hence, it is hard to examine whether there are any physician- or caregiver-related factors that affected the congruence. Although we encouraged the physicians to return their evaluations immediately after the encounters, on a few occasions, they returned it a few days later. Filling and returning the questionnaires late poses greater risks of recall error.

### 4.2. Clinical Implications

For clinicians, it is necessary to notice several threats to the quality of HRQOL discussions and evaluations, such as ignorance of certain symptoms, unclear priorities, and unawareness of the discrepancies of evaluation. Some patients with special characteristics, such as older age, lower educational level, or severe functional impairment, may need additional attention as their problems are at greater risk of being underestimated. In addition, when patients have a limited ability to report their problems, healthcare providers can consider caregivers to be reasonable sources for providing accurate but overestimated evaluation. Since social and financial problems are very complicated and often missed by physicians, patients may benefit from an inter-professional approach, such as partnering with nurses to facilitate discussions.

## 5. Conclusion

Congruence in symptom reports is a vital issue that has been attracting scientists and clinicians' attention for decades. Our study adds to the existing knowledge, as we addressed an Asian population, which has rarely been researched by similar studies. Since symptom management and communication can be quite different in diverse medical systems and cultures, the results of our study are valuable references for handling patients with similar backgrounds or in comparable settings. To the best of our knowledge, the current research is one of the few that closely monitors the congruence level of symptom reports after clinical encounters—collecting physicians' evaluations immediately after the OPD discussion enabled us to not only comprehend the quality of proxy ratings but also have a glimpse of the communication quality. Further research is needed to explore healthcare providers' perspective-taking methods and visualize the aforementioned concepts by linking them to patient outcomes. As congruence can be promoted [41], strategies that can facilitate symptom discussion and advance patient–physician congruence, such as using a symptom report instrument [42] or providing training sessions, are worth investigating.

## 6. Disclosure

This manuscript was submitted as a preprint in the link “https://www.medrxiv.org/content/10.1101/2020.09.29.20204404v1.”

## Figures and Tables

**Table 1:** Prevalence and intensity of symptoms, functioning status, and quality of life as rated by patients, physicians, and caregivers.

	Median score (interquartile range)		
	Patient	Doctor	Caregiver
Overall quality-of-life^1^	50 (33.3–66.7)	41.7 (31.3–66.7)	41.7 (25–62.5)
Functional domain			
Social	66.7 (33.3–100)	66.7 (33.3–100)	66.7 (33.3–83.3)
Physical	**73.3** *∗* (60–86.7)	**73.3** *∗* (51.7–86.7)	**73.3** *∗* (33.3–90)
Role	83.3 (50–100)	66.7 (33.3–100)	66.7 (25–100)
Emotion	83.3 (66.7–95.8)	75 (50–100)	75 (62.5–83.3)
Cognitive	83.3 (66.7–100)	83.3 (66.7–100)	83.3 (66.7–83.3)
Symptom domain			
Dyspnea	0 (0–33.3)	0 (0–33.3)	**33.3** *∗* (0–50)
Pain	25 (16.7–66.7)	**33.3** *∗* (0–66.7)	**33.3** *∗* (33.3–66.7)
Insomnia	33.3 (0–66.7)	33.3 (0–66.7)	**66.7** *∗* (33.3–83.3)
Fatigue	**44.4** *∗* (22.2–77.8)	33.3 (30.6–66.7)	**44.4** *∗* (33.3–88.9)
Appetite	33.3 (0–66.7)	33.3 (0–66.7)	33.3 (33.3–66.7)
Nausea/vomiting	**8.3** *∗* (0–33.3)	**16.7** *∗* (0–33.3)	0 (0–33.3)
Constipation	0 (0–33.3)	33.3 (0–33.3)	33.3 (0–33.3)
Diarrhea	0 (0–33.3)	0 (0)	0 (0–33.3)
Financial difficulties	**33.3** *∗* (0–33.3)	0 (0–66.7)	**33.3** *∗* (0–33.3)

*Note.* ^*∗*^Median scores reached clinical significance^33^; ^1^higher score means better situation in QOL and functioning but severer problems in symptoms.

**Table 2:** Underestimation and overestimation of symptoms, functioning, and health-related quality of life by physicians and caregivers as compared to patients.

Physician ratings compared to patient ratings (*n* = 45–52 pairs^1^)												Symptoms, functioning impairment, and health related quality of life	Caregiver ratings compared to patient ratings (*n* = 25–27 pairs^1^)											
Physician underestimation					Complete agreement		Physician overestimation						Caregiver underestimation					Complete agreement		Caregiver overestimation^2^				
−**4**	−**3**	−**2**	−**1**	**(%)**	**0**	**(%)**	**+1**	**+2**	**+3**	**+4**	**(%)**		−**4**	−**3**	−**2**	−**1**	**(%)**	**0**	**(%)**	**+1**	**+2**	**+3**	**+4**	**(%)**
6	5	4	9	46.2	8	15.4	4	11	2	3	39.5	Health related quality of life	2	2	5	3	44.4	7	25.9	4	3	1	0	29.6
4	2	11	5	45.8	11	22.9	5	4	2	4	31.3	Social functioning	1	1	5	4	40.7	7	25.9	2	3	1	3	33.3
6	5	5	7	44.2	8	15.4	9	7	1	4	40.4	Fatigue	0	0	3	7	37	4	14.8	7	2	2	2	48.1
0	1	1	18	40.8	11	22.4	14	3	1	0	36.7	Insomnia	0	0	0	6	22.2	9	33.3	9	2	1	0	44.4
1	2	6	11	38.5	17	32.7	10	1	3	1	28.8	Pain	0	1	0	3	14.8	7	25.9	8	5	1	2	59.2
0	1	5	12	34.6	18	34.6	12	3	1	0	30.8	Appetite loss	0	0	1	7	29.6	14	51.9	1	3	1	0	18.5
0	0	1	11	23.1	37	71.1	3	0	0	0	5.8	Diarrhea	0	0	0	5	18.5	16	59.2	6	0	0	0	22.2
0	0	3	6	17.3	29	55.8	7	7	0	0	26.9	Constipation	0	0	0	5	18.5	19	70.4	3	0	0	0	11.1
0	1	4	11	33.3	25	52	6	1	0	0	14.6	Financial difficulties	0	0	2	2	14.8	14	51.9	8	1	0	0	33.3
0	0	2	45	26.9	24	46.1	10	3	1	0	26.9	Dyspnea	0	0	1	2	11.1	15	55.6	5	3	1	0	33.3
1	2	3	10	34	21	44.7	7	3	0	0	21.3	Cognitive functioning	0	0	2	2	14.8	13	48.1	6	1	3	0	37
1	0	4	8	25	20	38.5	7	7	2	3	36.5	Nausea and vomiting	0	0	4	3	25.9	12	44.4	5	1	1	1	29.6
0	3	8	3	28	12	24	5	11	2	6	48	Role functioning	0	0	2	3	19.2	10	38.5	3	5	0	3	42.3
4	3	5	6	40	8	17.8	6	1	4	8	42.2	Physical functioning	2	0	1	2	20	5	20	6	5	0	4	60
4	3	7	4	37.5	10	20.8	7	2	3	8	41.7	Emotional functioning	1	1	2	2	23.1	5	19.2	3	5	3	4	57.7

*Note.* The comparison of the dyad's rating was counted by sum score differences (caregiver score minus patient score) on functional impairment/symptoms/health-related quality of life. The grey area indicates where the majority of proxies underestimate, overestimate, or agree with the patients. ^1^The pairs were eliminated if either patient, physician, or caregiver in that pair has an incomplete data. The original eligible pairs were 52 pairs for patient-physician dyads and 27 pairs for patient-caregiver dyads. ^2^Overestimated for all items suggest that the proxy rated the problem severer than the patient, except for health-related quality of life ratings (overestimated for health related quality of life means that proxy perceived a better condition than patient's perception).

## Data Availability

The data that support the findings of this study are available on request from the corresponding author due to them containing information that could compromise the privacy of research participants.
